# *Clostridium difficile* toxins or infection induce upregulation of adenosine receptors and IL-6 with early pro-inflammatory and late anti-inflammatory pattern

**DOI:** 10.1590/1414-431X20209877

**Published:** 2020-07-24

**Authors:** D.A. Foschetti, M.B. Braga-Neto, D. Bolick, J. Moore, LA. Alves, CS. Martins, LE. Bomfin, AAQA. Santos, RFC. Leitão, GAC. Brito, CA. Warren

**Affiliations:** 1 Departamento de Morfologia, Faculdade de Medicina, Universidade Federal do Ceará, Fortaleza, CE, Brasil; 2 Division of Infectious Diseases and International Health, University of Virginia, Charlottesville, VA, USA; 3 Departamento de Ciências Médicas, Faculdade de Medicina, Universidade Federal do Ceará, Fortaleza, CE, Brasil

**Keywords:** Clostridium difficile, *Clostridium difficile* toxins, Adenosine, A_2A_ adenosine receptor, A_2B_ adenosine receptor

## Abstract

*Clostridium difficile* causes intestinal inflammation, which increases adenosine. We compared the expression of adenosine receptors (AR) subtypes A_1_^, A^_2A_^, A^_2B_^, and A^_3_ in HCT-8, IEC-6 cells, and isolated intestinal epithelial cells, challenged or not with *Clostridium difficile* toxin A and B (TcdA and TcdB) or infection (CDI). In HCT-8, TcdB induced an early A_2B_R expression at 6 h and a late A_2A_R expression at 6 and 24 h. In addition, both TcdA and TcdB increased IL-6 expression at all time-points (peak at 6 h) and PSB603, an ^A^_2B_R antagonist, decreased IL-6 expression and production. In isolated cecum epithelial cells, TcdA induced an early expression of ^A^_2B_R at 2s and 6 h, followed by a late expression of A_2A_R at 6 and 24 h and of A_1_R at 24 h. In CDI, A_2A_^R and A^_2B_R expressions were increased at day 3, but not at day 7. ARs play a role in regulating inflammation during CDI by inducing an early pro-inflammatory and a late anti-inflammatory response. The timing of interventions with AR antagonist or agonists may be of relevance in treatment of CDI.

## Introduction

*Clostridium difficile* (*C*. *difficile*) is a major cause of antibiotic-associated diarrhea in hospitalized patients (1). Since the early 2000’s, considerable changes in the epidemiology and severity of *C*. *difficile* infection (CDI) have been observed worldwide, which has been related to the rise of more virulent strains such as NAP1/B1/027 (2–4).

This anaerobic bacterium produces two major exotoxins, toxin A (TcdA) and toxin B (TcdB), both with glucosyltransferase activity, which permanently inactivates Rho GTPases causing disaggregation of actin cytoskeleton, activation of caspases, and intestinal cell damage (5,6). *In vitro,* both TcdA and TcdB decrease intestinal cell migration and proliferation and induce apoptosis by activation of extrinsic and intrinsic apoptosis pathways (7–9). We have previously demonstrated that TcdA attenuates Wnt/ß-catenin signaling in intestinal epithelial cells, which is associated with anti-proliferative effects (10). In animal models, these toxins also cause intestinal secretion, intense destruction of the mucosa, hemorrhage, and accentuated tissue inflammation with neutrophil infiltration and production of cyclooxygenase-2, prostaglandin E2, and inflammatory cytokines such as tumor necrosis factor (TNF)-*α* and interleukin (IL)-1β, IL-6, and IL-8 (11,12). Treatment of CDI still relies on antimicrobial agents such as vancomycin or fidaxomycin (13). Unfortunately, antimicrobial therapy may create a susceptible environment for reinfection or relapse by disrupting the gut microbial flora (14). Furthermore, a subset of patients can be refractory to available medical therapy, including fecal transplant, highlighting the need for novel treatment options.

Adenosine, an endogenous purine nucleoside, accumulates in the extracellular space during stressful conditions, such as ischemia, hypoxia, and inflammation, and modulates the immune and inflammatory responses (15). Adenosine elicits its effects through four transmembrane adenosine receptors: A_1_^, A^_2A_^, A^_2B_, and ^A^_3_, which all act on mitogen-activated protein kinase pathways (MAPK) (^16^). Receptors ^A^_1_ and ^A^_3_ increase concentration of calcium, while receptors ^A^_2A_ and ^A^_2B_ increase cyclic AMP. While activation of receptor ^A^_2A_ induces an anti-inflammatory response, activation of receptor ^A^_2B_ is associated with a pro-inflammatory response (17,18). Indeed, we have demonstrated *in vitro* and *in vivo* that ^A^_2A_ agonists or ^A^_2B_ antagonist can ameliorate *C. difficile* colitis (19,20). Interestingly, the expression, distribution, and co-localization of these receptors in the gastrointestinal tract intestine varies between cell types (21), highlighting the importance of investigation of the cell-specific roles of ^A^_2A_^R and A^_2B_^R.^

In this study, we evaluated the expression of AR specifically in isolated cecum epithelial cells following CDI or exposure to TcdA and found a time-dependent expression pattern of A_2B_ and A_2A_. Similar results were observed *in vitro* following exposure to TcdA and TcdB and correlated with expression of IL-6, a pro-inflammatory cytokine.

## Material and Methods

### Cell culture

A human ileocecal epithelial cell line, HCT-8 cells (passages 20–30), were grown in filtered RPMI medium 1640 in the presence of 10% fetal bovine serum, 1 mM sodium pyruvate, and 0.1 unit/mL of penicillin/streptomycin (Gibco, cat #15140, USA). Rat intestinal jejunal crypt cells, IEC-6 cells (passages 17–30), were grown in Dulbecco's modified Eagle's medium (DMEM) supplemented with 10% fetal bovine serum (Gibco), 1 mM sodium pyruvate, 95% bovine insulin, and 0.1 unit of pen/strep. All cells were maintained in a humidified incubator at 37°C and 5% CO_2_. ^Trypsin-EDTA-^dissociated HCT-8 cells, in 200 µL of the medium, were seeded in a 6-well plate. Upon 80% confluence, the cells were treated with TcdA or TcdB (0.01, 0.1, 1, 10, and 100 ng/mL) and were incubated for 2, 6, and 24 h.

### Murine cecal injection model

We performed the murine cecal injection as previously described (22). This protocol was approved by the Center for Comparative Medicine at the University of Virginia (USA). C57BL/6 male mice, weighing 23–25 g each, were fasted overnight. The mice were anesthetized with ketamine (60–80 mg/kg) and xylazine (5–10 mg/kg), administered intramuscularly. A midline abdominal incision was made to expose the cecum. After flushing with PBS, 20 μg of toxin A in 100 μL of 0.9% normal saline was injected into the distal tip. Incisions were sutured (nylon 3-0, Procare, Brazil) (time 0) for 2, 6, or 24 h and animals were monitored during recovery. Sham-injected animals received only 100 μL of saline and animals were monitored during recovery. Any moribund (i.e., hunched posture, ruffled coat, or little to no movement) mouse was immediately euthanized. In animal studies, TcdA appears to be the dominant virulence factor compared to TcdB (23,24). Therefore, we chose to use TcdA, not TcdB, in the murine model.

### Isolation of cells from cecal tissue

The cecum epithelial cells isolation protocol was followed according D'Auria et al. (22). A cross-section from the middle of each cecum was dissected and opened longitudinally, rinsed with Hank's balanced salt solution (HBSS; Gibco), and shaken at 250 rpm for 30 min at 37°C in HBSS containing 50 mM EDTA and 1 mM dithiothreitol (DTT) in order to remove epithelial-layer cells. The digested tissue was strained with a 100-μm cell strainer and the filtrate was centrifuged (1,000 *g*, 4°C, 10 min). Cells were resuspended in red-cell lysis buffer (150 mM NH_4_Cl, 10 mM NaHCO_3_, 0.1 mM EDTA) and centrifuged again. The pelleted cells were stored at −80°C for further RNA isolation and cytokine quantification.

### Murine model of *C. difficile* infection

The infection model was a modification of a previously described protocol (25). This protocol has been approved by the Center for Comparative Medicine at the University of Virginia. From 6 to 4 days prior to infection, C57BL/6 mice were given an antibiotic cocktail containing vancomycin (0.0045 mg/g), colistin (0.0042 mg/g), gentamicin (0.0035 mg/g), and metronidazole (0.0215 mg/g) in drinking water. One day prior to infection, clindamycin (32 mg/kg) was injected subcutaneously. Infection was performed with strain VPI 10463 at an inoculum of 10^5^ cells administered by oral gavage. The uninfected control group received only the vehicle. A group of infected and uninfected mice were sacrificed by cervical dislocation under sedation (ketamine-xylazine) on day 3 and at the end of the experiment (day 7). Cecal tissues were harvested and frozen until mRNA extraction and AR gene expression assay were performed.

### Adenosine receptor subtype assay

^Adenosine receptor subtype (A^_1_^, A^_2A_^, A^_2B_^, and A^_3_), was assayed by quantitative PCR (qPCR) in IEC-6, HCT-8, or in mouse cecum epithelial cells. Purified TcdA and TcdB were provided by David Lyerly from TECHLAB, Inc. (USA). Each sample was suspended in 350 μL of RLT lysis buffer and the RNA was extracted using Qiagen RNeasy mini kit (USA), according to manufacturer's instructions. RNA was quantified by standard spectrophotometry (Biophotometer, Eppendorf, Germany). In order to remove the genomic DNA carried over from RNA extraction, DNase I (Ambion, USA) treatment was performed following the manufacturer's instructions. Synthesis of cDNA by reverse transcriptase PCR was performed using SuperScript III First-Strand Synthesis System SuperMix (Invitrogen, USA) with the use of oligo (dT) as primers. cDNA was used in quantitative PCR for measuring A_1_^, A^_2A_, ^A^_2B_, and ^A^3 expression compared to GAPDH expression. The Invitrogen Fast SYBR green cells-to-CT one-step kit was used according to the manufacturer's instructions, as previously described (26). The relative gene expression was determined using the 2−ΔΔCt (25) method using GAPDH as the housekeeping gene.

### Cytokine gene assay

Total cellular RNA extraction from each intestinal tissue, analysis, cDNA conversion, and qPCR protocol are described above (26,27). The primers used for both adenosine subtype and cytokine gene expression are listed on Table 1.

**Table 1:** List of primer sequences for reverse transcription-qPCR analyses.

Gene	Primers	Sequence
A_1_ AR	Forward	GCGGTGAAGGTGAAC
	Reverse	AGGCAGGTGTGGAAG
A_2A_ AR	Forward	AGTTCCGCCAGACCTTCC
	Reverse	AGTTCCGCCAGACCTTCC
A_2B_ AR	Forward	GGTCATTGCTGTCCTCTG
	Reverse	CAGGTGAGCCAGCAAGATC
A_3_ AR	Forward	AGGGTAGGAATGAGCAAGTTG
	Reverse	CAGGTGAGCCAGCAAGATC
GAPDH	Forward	AGGTCGGAGTCAACGGATTTGGT
	Reverse	CATGTGGGCCATGAGGTCCACCAC
IL-6	Forward	ACAAGTCGGAGGCTTAATTACACAT

### Cytokine quantification by ELISA

IL-6 concentrations in cecum tissue were measured by enzyme-linked immunosorbent assay (ELISA) as described previously (28).

### Immunohistochemical reaction for IL-6

Immunohistochemistry (IHC) for IL-6 was performed in cecum tissue using the streptavidin-biotin-peroxidase method (29) in formalin-fixed, paraffin-embedded tissue sections (4-μm thick) mounted on poly(l)-lysine-coated microscope slides. Sections were incubated overnight (4°C) with primary rabbit anti-mouse IL-6 (Santa Cruz Biotechnology, USA) in PBS plus bovine serum albumin (PBS-BSA). The slides were then incubated with biotinylated goat anti-rabbit IgG and diluted in PBS-BSA. After being washed, the slides were incubated with avidin-biotin-horseradish peroxidase conjugate (ABC complex; Santa Cruz Biotechnology) for 30 min according to the manufacturer’s protocol. IL-6 was visualized with chromogen 3,3'diaminobenzidine (DAB). Negative-control sections were processed simultaneously as described above but with the first antibody being replaced by PBS-5% BSA. Slides were counterstained with Harris hematoxylin (Dinâmica, Brazil).

### Statistical analysis

Data are reported as means±SE, as generated by GraphPad Prism version 5.0 (GraphPad Software, USA). The differences between experimental groups were evaluated using one-way analysis of variance (ANOVA) with Bonferroni's multiple comparison test. Student's *t*-test was performed to analyze differences between 2 groups. Statistical significance was set at P≤0.05.

## Results

### A_2B_R was the predominant AR expressed in intestinal epithelial cells

**Figure 1:**
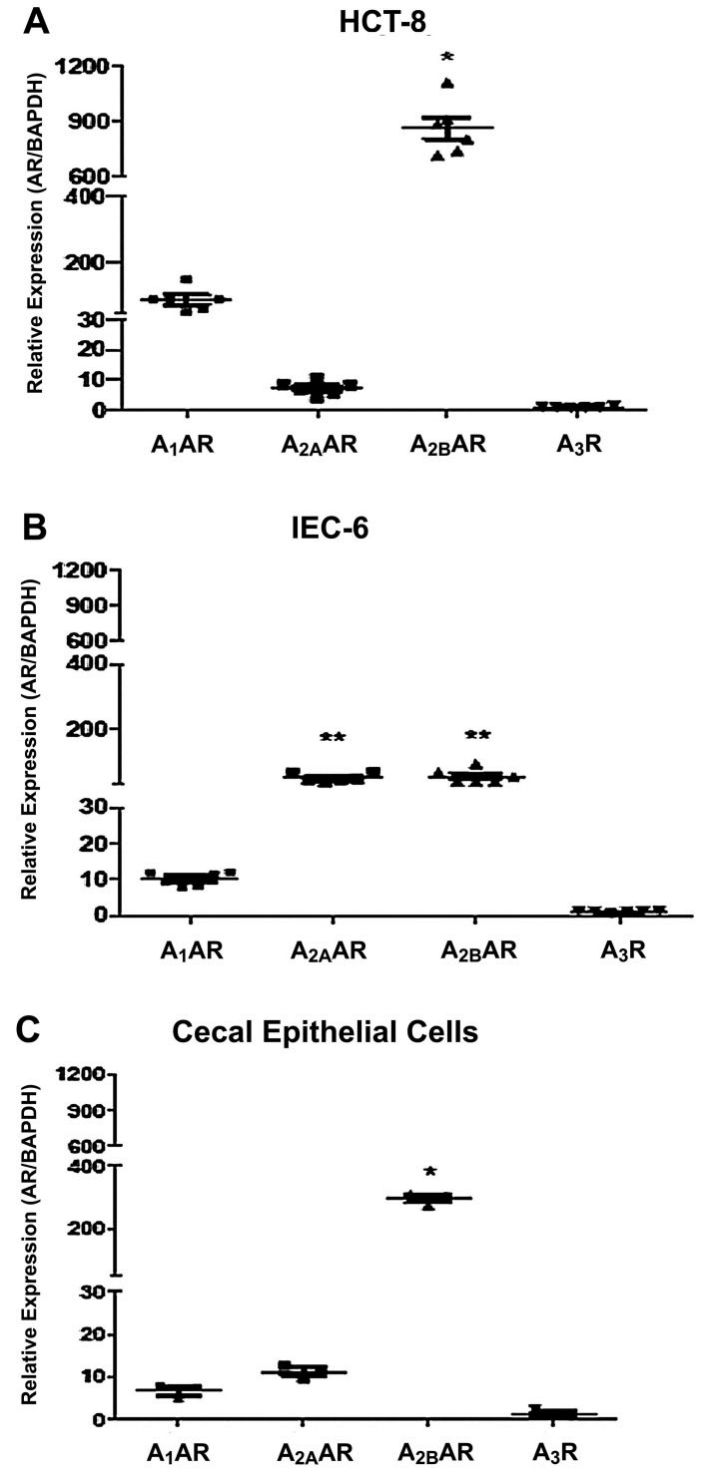
Adenosine receptor (AR) gene expression in intestinal human (HCT-8) and rat (IEC-6) cells and isolated cecal epithelial cells. HCT-8 (**A**) and IEC-6 (**B**) cells were incubated with specific media and, after achieving confluence, cells were harvested and mRNA were extracted and analyzed by qPCR. **C**, The cecum epithelial cells from mice (n=6 per group) were isolated and the mRNA was extracted for A_1_^AR, A^_2A_^AR, A^_2B_^AR, and A^_3_AR analysis by qPCR. Each assay was performed in triplicate per time-point. *P<0.05, compared with A_1_^AR, A^_2A_^AR, and A^_3_AR; **P<0.05, compared with A_1_^AR and A^_3_AR (one-way ANOVA with Bonferroni post-test). Vertical lines indicate mean±SE.

The mean of A_2B_ mRNA expression was 10-fold higher (P<0.05) than that of A_1_ and >150-fold higher (P<0.05) than those of A_2A_ and A_3_ transcripts in HCT8 cells at baseline (Figure 1A). However, in IEC-6 cells, both A_2A_ and A_2B_ mRNAs were significantly more expressed than A_1_ and A_3_ (Figure 1B). In cecum epithelial cells isolated from healthy mice, mRNA levels of A_2B_ were significantly higher than all other ARs followed by A_2A_, as shown in Figure 1C. Therefore, for our *in vitro* experiments of TcdA and TcdB intoxication, HCT-8 cells were used, as its adenosine receptor pattern more closely resembled cecal epithelial cells compared to IEC-6 cells.

### TcdA and TcdB upregulated AR expression in HCT-8 cells

**Figure 2:**
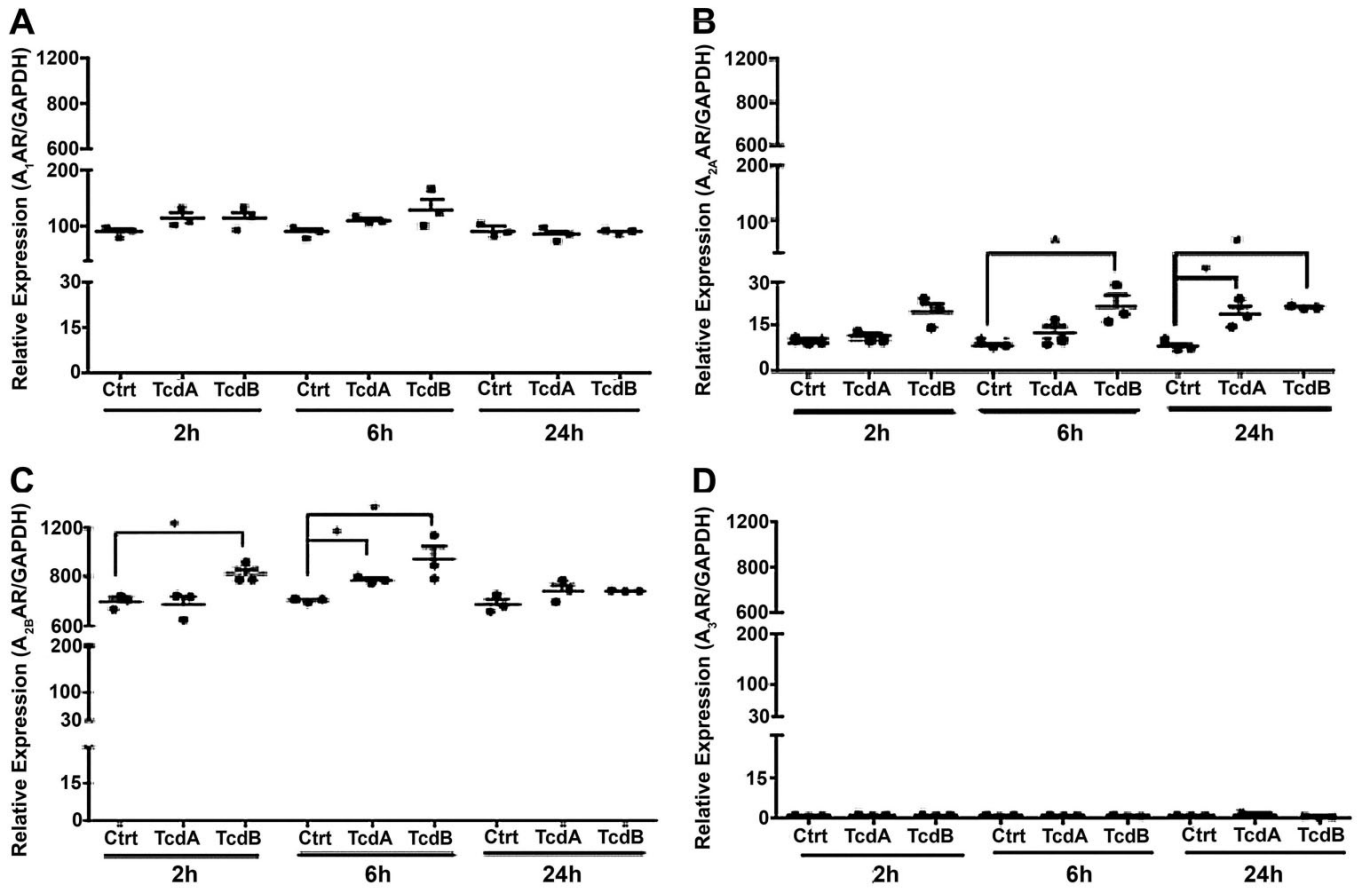
Effect of *C. difficile* toxins on adenosine receptor (AR) expression *in vitro*. HCT-8 cells were intoxicated with TcdA or TcdB (10 ng/mL) for 2, 6, and 24 h. Analyses of A_1_AR (**A**), A_2A_AR (**B**), A_2B_AR (**C**), and A_3_AR (**D**) mRNA expression were performed by qPCR. Each assay was performed in triplicate per time-point. *P<0.05 compared with control (Ctrl) (one-way ANOVA with Bonferroni post-test). Vertical lines indicate mean±SE.

To test whether *C. difficile* toxins affect AR expression *in vitro*, we incubated HCT-8 cells with TcdA or TcdB. A_2B_ mRNA significantly increased after 2 and 6 h of exposure to 10 ng/mL TcdB (Figure 2C). A_2A_ mRNA significantly increased after 6 and 24 h of exposure to TcdB (Figure 2B). TcdA at 10 ng/mL significantly increased A_2B_ and A_2A_ transcript expression after 6 h and 24 h of exposure, respectively. There was no significant difference in A1 or A3 mRNA expression in response to TcdA and TcdB (Figure 2A and D).

### *C. difficile* toxin-induced IL-6 secretion was mediated by A_2B_R

**Figure 3:**
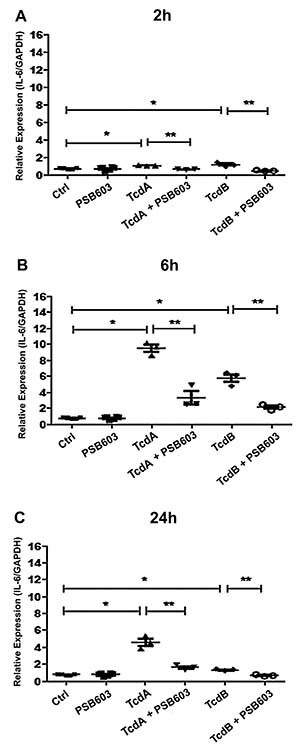
Effect of A_2B_AR antagonist (PSB603) on *C. difficile*-induced interleukin (IL)-6 gene expression *in vitro.* HCT-8 cells were incubated with TcdA and TcdB at 10 ng/mL with or without PSB603. Analyses of IL-6 mRNA expression at 2 (**A**), 6 (**B**), and 24 (**C**) h were performed by qPCR. Each treatment was done in triplicate per time-point. *P<0.05, **P<0.05 (one-way ANOVA with Bonferroni post-test). Vertical lines indicate mean±SE.

Because both TcdB and TcdA predominantly induced the expression of A_2B_ in HCT-8, we investigated whether this was associated with IL-6 gene expression by using PSB603, a specific A_2B_ antagonist. TcdB increased IL-6 gene expression by 1.6-, 7.4-, and 1.6-fold at 2, 6, and 24 h, respectively (Figure 3).

Incubation with A_2B_ antagonist, PSB603, significantly decreased IL-6 secretion at 2, 6, and 24 h. Consistent with the timing of peak A_2B_ expression, IL-6 gene expression also peaked at 6 h with TcdB stimulation.

### TcdA and *C. difficile* infection induced AR expression in isolated cecum epithelial cells

**Figure 4:**
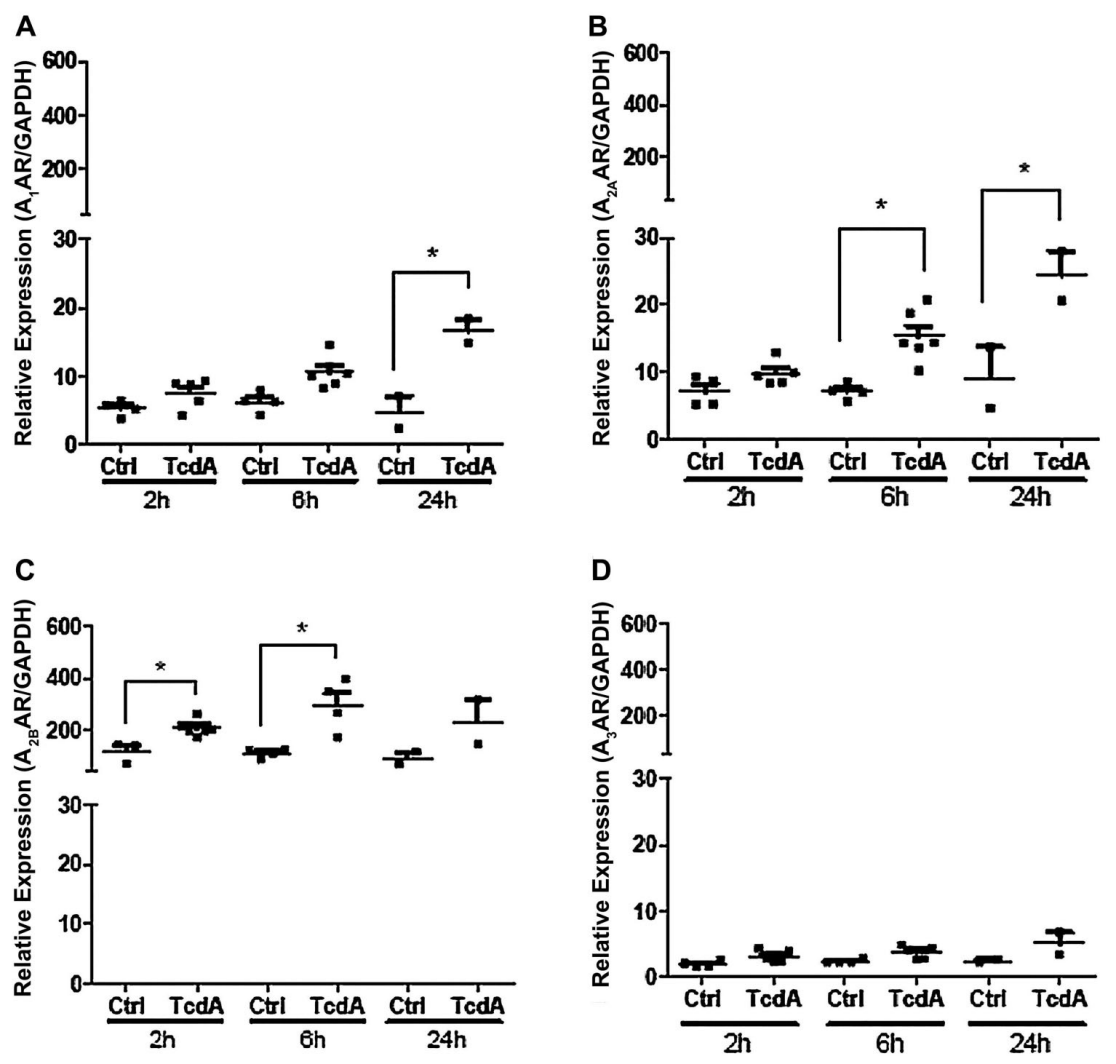
Effects of *C. difficile* TcdA on the adenosine receptors (AR) gene expression in cecal epithelial cells. The murine cecum (n=6/group) was injected with TcdA (20 µg/loop) and incubated for 2, 6, and 24 h. The cecal epithelial cells were isolated and mRNA was extracted for A_1_^AR (**A**), A^_2A_^AR (**B**), A^_2B_^AR (**C**), andA^_3_^AR^ (**D**) analysis by qPCR. *P<0.05 compared with control (Ctrl) (one-way ANOVA with Bonferroni post-test). Vertical lines indicate mean±SE.

**Figure 5:**
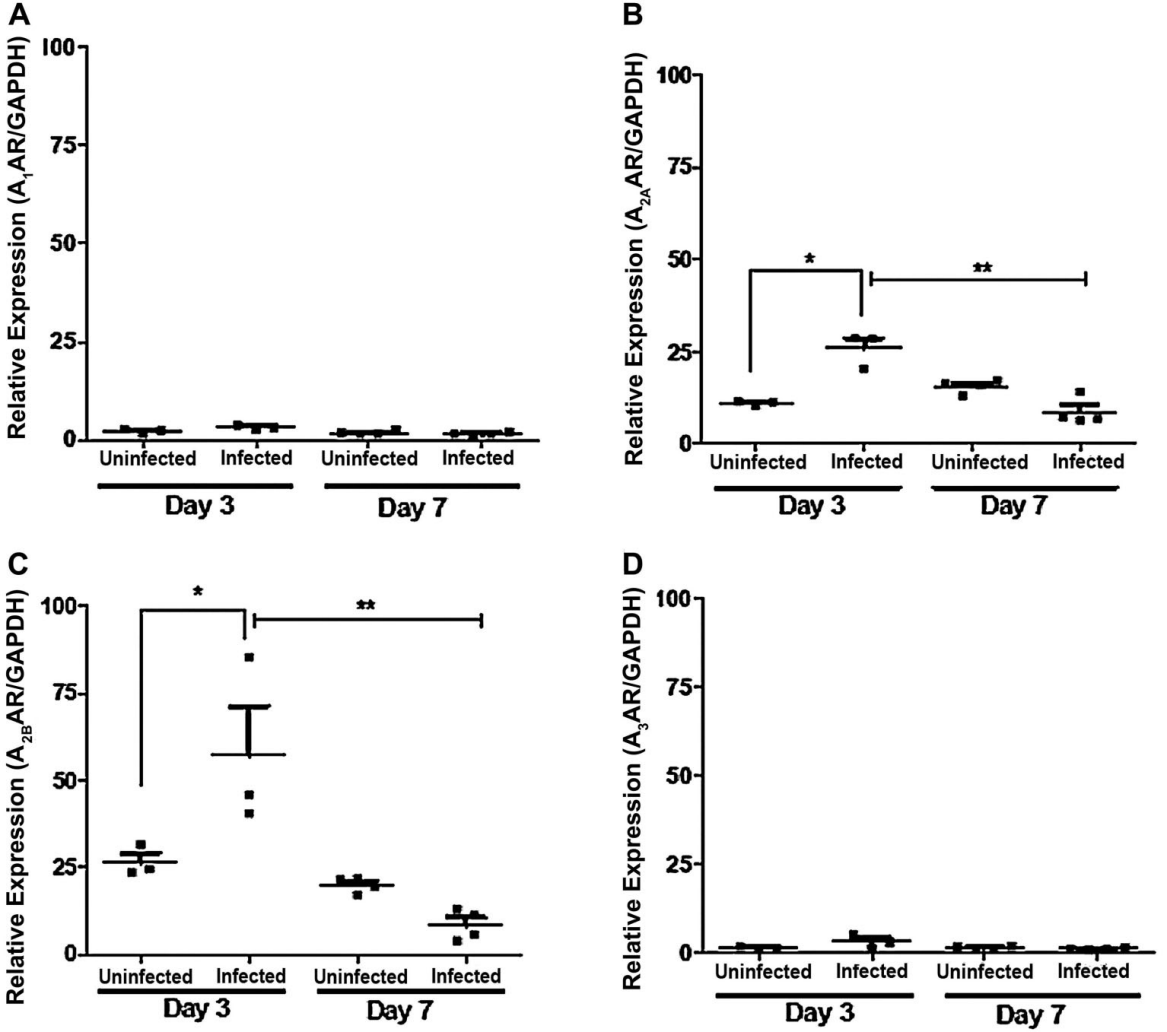
Effects of *C. difficile* infection on the AR in cecal tissue. Mice (n=3 to 4/group) were infected with *C. difficile* (10^5^ CFU, colony forming units) and were sacrificed at either 3 or 7 days after the infection. mRNA was extracted from cecal tissues for A_1_^AR (**A**), A^_2A_^AR (**B**), A^_2B_^AR^ (**C**), and A_3_^AR^ (**D**) analysis by qPCR. *P<0.05 compared with uninfected mice; **P<0.05 compared with infected mice at day 3 (one-way ANOVA with Bonferroni post-test). Vertical lines indicate mean±SE.

To test the effect of *C. difficile* toxins in AR expression *in vivo*, we injected mouse cecal loops with TcdA as we had previously demonstrated that TcdA, and not TcdB, induced consistent histopathological findings in both mouse and rabbit intestinal tissues (28,29). After 2, 6, and 24 h of exposure, epithelial cells isolated from cecal tissues ^challenged with TcdA had significantly higher mRNA levels of A^_1_, ^A^_2A_, and ^A^_2B_ subtypes compared to their respective controls (Figure 4). ^A^_2B_R subtype expression significantly increased at 2 and 6 h (Figure 4C), ^A^_2A_R at 6 and 24 (Figure 4B), and ^A^_1_ only at 24 h (Figure 4A). No significant difference in ^A^_3_R mRNA expression levels was observed (Figure 4D). Again, ^A^_2B_ levels were the most highly expressed amongst the AR subtypes, with peak expression at 6 h of incubation. To evaluate the effect of C. *difficile* infection on AR subtype expression, we harvested cecal tissues from infected mice at days 3 (infection peak) and 7 (recovery period) post-infection. Cecal tissues harvested at day 3 post-infection with *C. difficile* had higher ^A^_2A_ and ^A^_2B_ mRNA expressions compared to their respective uninfected controls (Figure 5A-D). At day 7 post-infection, no significant differences in ^A^_2A_ and ^A^_2B_ mRNA expressions were observed compared to uninfected controls but there was a significant decrease in both ^A^_2A_ and ^A^_2B_ mRNA expressions at day 7 compared to infected mice at day 3. No significant differences regarding mRNA levels of ^A^_3_ were observed among the groups (Figure 5D)

### *C. difficile* toxin-induced IL-6 secretion decreased with the A_2B_R blockage *in vivo*

**Figure 6:**
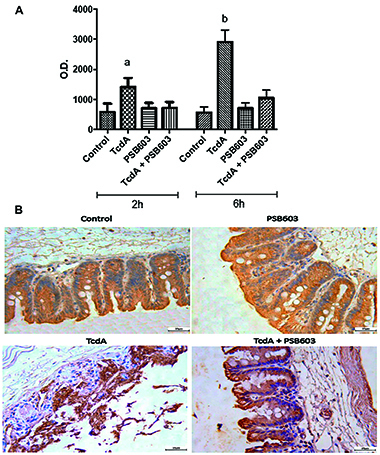
Effect of A_2B_AR antagonist (PSB603) on *C. difficile*-induced interleukin (IL)-6 production *in vivo.* The murine cecum (n=6/group) was injected with TcdA (20 µg/loop) with or without PSB603 (5 µM) and incubated for 2 and 6 h. The cecum epithelial cells were isolated and IL-6 production was detected by ELISA and reported as absorbance (O.D.) units (**A**). ^a^P<0.05 compared with Ctrl 2 h; ^b^P<0.05 compared with Ctrl 6 h (one-way ANOVA with Bonferroni post-test). Data are reported as mean±SE. **B**, The presence of IL-6 in the enterocytes from cecal tissues was detected by immunohistochemistry. Representative tissues shown were harvested at 6 h (scale bar 25 μm).

IL-6 concentration in murine cecal epithelial cells intoxicated with TcdA was evaluated by ELISA and IHC. After 2 h of exposure, there was no difference in IL-6 production. However, 6 hours after TcdA incubation, IL-6 production and immunoreactivity increased significantly in cecal enterocytes compared to the control group (Figure 6A and B). The animals ^intoxicated with TcdA and treated with the A^_2B_^antagonist PSB603 had significantly^ lower IL-6 levels and immunoreactivity than untreated mice.

## Discussion

In this study, we demonstrated for the first time the expression of AR subtypes specifically in isolated cecal epithelial cells in a murine model of CDI or TcdA intoxication and identified a distinct expression pattern during early and late infection, contributing to the understanding of the cell-specific pathogenesis of CDI. Indeed, we also confirmed *in vitro* using a human intestinal cell line, HCT-8, that ^A^_2B_^expression increased at earlier time-points of intoxication while A^_2A_ increased at later time-points.

Previously, we have demonstrated the effect of TcdA and TcdB on the expression of adenosine receptors after 2 and 4 h of intoxication in HCT-8 cells (19). The present work used short-term (2 and 6 h) and, importantly, long-term incubation with TcdA or TcdB (24 h), demonstrating the effect on adenosine receptors both *in vitro* (HCT-8 cells) and *in vivo* (cecal epithelial cells), and the effect of infection with the microorganism on adenosine receptors. The short-term effects of toxins A and B on the expression of adenosine receptors in HCT-8 cells demonstrated in the current study corroborated previously published findings, suggesting that intestinal epithelial cells upregulate A_2B_ AR mRNA expression in response to *C*. *difficile* toxins (19).

We have previously shown that the A_2A_R agonist, ATL313, significantly decreased intestinal damage and TNF-α production induced by *C. difficile* TcdA in mice (20). A subsequent study combining A_2A_R agonist therapy (ATL370) with alanlyl-glutamine supplementation demonstrated improvement of intestinal damage and increased IL-10 levels during TcdA intoxication (19). In the present study, by isolating the cecal epithelial, cells *in vivo*, we were able to evaluate separately the effect of TcdA on adenosine receptor expression and have found that A_2B_ is the most highly expressed amongst the AR subtypes. In fact, according to the literature, A_2A_ may be more localized in immune rather than epithelial cells in the intestinal tract (30). In accordance, in macrophages, A_2A_ receptor activation decreases secretion of inflammatory cytokines, such as TNF-α and IL-6 and increases IL-10 (31,32).

Several studies have confirmed the ^A^_2B_R pro-inflammatory role by demonstrating that ^A^_2B_R blockade with selective antagonists decreases IL-6 levels and neutrophil activation, resulting in decreased intestinal damage in mice suffering from colitis (33,34) or infected with *C. difficile* (19). In HCT-8 cells and cecum epithelial cells, TcdA- or TcdB-induced IL-6 secretion is significantly decreased by ^A^_2B_^R blockade. Additionally, it was seen that blocking or knockdown of A^_2B_^R caused a significant decrease in IL-6 secretion by the enterocytes and^ submucosal cells in infected animals, suggesting that the expression of pro-inflammatory cytokines such as IL-6 by intestinal epithelial cells are induced via ^A^_2B_R stimulation and activation of its intracellular signaling pathway (19).

In this study, we evaluated the effect of TcdA on AR subtype expression specifically in isolated mouse cecal epithelial cells. Although both TcdA and TcdB are important for pathogenesis, we did not use TcdB in our murine model experiments since it has been previously shown that rabbits, hamsters, and mice are more responsive to TcdA, compared with TcdB (35–37). TcdA increased the expression of all AR subtypes. Specifically, TcdA induced an early peak (2 and 6 h) of ^A^_2B_ and a late peak (6 and 24 h) of ^A^_2A_. We also found that ^A^_1R_ expression was increased at 24 h. Adenosine ^A^_1_ is known to have predominantly a pro-inflammatory effect. However, since ^A^_2B_ was shown to be the predominant AR expressed in cecal epithelial cells, we believe that the overall inflammatory state was more closely linked to ^A^_2B_ expression.

Using a murine model of CDI, we isolated the intestinal cecum epithelial cells in mice at days 3 and 7 post-infection with *C. difficile* and assessed the AR subtype expression pattern. We found that infection increased the expression of ^A^_2A_ and ^A^_2B_ at day 3, compared to uninfected controls, with a predominance of ^A^_2B_. During the infection recovery (day 7), expression of both receptors was significantly decreased. These findings are consistent with our previous studies suggesting the potential critical role of ^A^_2B_ activity in the pathogenesis of CDI (19) and support the role of ^A^_2A_ in controlling inflammation-induced damage.

Considering that HCT8 is a human cell line and more closely resembled the AR subtype pattern observed in isolated cecal epithelial cells *in vivo* compared to rat intestinal epithelial cells, we chose this cell line to evaluate the effect of TcdA and TcdB over time on the expression of AR subtypes *in vitro*. Pro-inflammatory cascade likely predominated at early time points of intoxication as supported by an early peak of ^A^_2B_R expression, followed later by the anti-inflammatory cascade as supported by a late peak of ^A^_2A_R expression. We hypothesized that, initially, there is a peak of pro-inflammatory cytokines, such as IL-6, in the intestinal epithelium that may result in the activation of macrophages and the recruitment of neutrophils to control infection. However, following the massive release of pro-inflammatory cytokines and production of free radicals, the intestinal epithelium possibly shifts towards an anti-inflammatory milieu to limit the tissue damage induced by exaggerated immune and pro-inflammatory responses. Similarly, dendritic cells when mature express higher levels of ^A^_2A_, switching from a pro- to an anti-inflammatory response, with increased levels of IL-10 and lower levels of IL-1 beta, TNF-α, and IFN-gamma (38–40) .

In conclusion, we demonstrated that *C. difficile* toxins upregulate predominantly A_2A_ and A_2B_ subtypes in the intestinal epithelium, with an early expression of A_2B_ and IL-6, followed by a late A_2A_ gene expression. A_2B_ appears to be critical for IL-6 gene expression and production in HCT8 and cecum epithelial cells. Similarly, in our infection model, A_2B_ seemed to be the predominant AR expressed during acute infection, which may partially explain the highly inflammatory feature of the *C. difficile-*associated diarrhea. Our findings provide insight into the sequence of events in adenosine receptor subtype expression upon exposure to toxins and the potential importance of timing of intervention to maximize potential beneficial outcomes of treatment following *C. difficile* infection.
